# Molecular changes in solitary fibrous tumor progression

**DOI:** 10.1007/s00109-019-01815-8

**Published:** 2019-07-18

**Authors:** Hyung Kyu Park, Dan Bi Yu, Minjung Sung, Ensel Oh, Mingi Kim, Ji-Young Song, Mi-Sook Lee, Kyungsoo Jung, Ka-Won Noh, Sungbin An, Kyoung Song, Do-Hyun Nam, Yu Jin Kim, Yoon-La Choi

**Affiliations:** 1 Department of Pathology, Konkuk University Medical Center, Konkuk University School of Medicine, Seoul, South Korea; 2 Department of Health Sciences and Technology, SAIHST, Sungkyunkwan University, Seoul, South Korea; 3 Laboratory of Cancer Genomics and Molecular Pathology, Samsung Medical Center, Sungkyunkwan University School of Medicine, Irwon-ro 81, Gangnam-gu, Seoul, 06351 South Korea; 4 The Center for Companion Diagnostics, LOGONE Bio Convergence Research Foundation, Seoul, South Korea; 5 Department of Pathology and Translational Genomics, Samsung Medical Center, Sungkyunkwan University School of Medicine, Irwon-ro 81, Gangnam-gu, Seoul, 06351 South Korea

**Keywords:** Solitary fibrous tumor, *NAB2-STAT6*, Molecular change, *APAF1*, *TP53*

## Abstract

**Abstract:**

Solitary fibrous tumors (SFTs) are *NAB2*-*STAT6* fusion-associated neoplasms. There are several subtypes of *NAB2-STAT6* fusions, but their clinical significances are still unclear. Moreover, the mechanisms of malignant progression are also poorly understood. In this study, using 91 SFT cases, we examined whether fusion variants are associated with clinicopathological parameters and also investigated the molecular mechanism of malignant transformation using whole-exome sequencing. We detected variant 1b (*NAB2*ex4-*STAT6*ex2) in 51/91 (56%) cases and variants 2a/2b (*NAB2*ex6-*STAT6*ex16/17) in 17/91 (19%) cases. The *NAB2-STAT6* fusion variant types were significantly associated with their primary site (*P* < 0.001). In addition, a *TERT* promoter mutation was detected in 7/73 (10%) cases, and it showed a significant association with malignant SFTs (*P* = 0.003). To identify molecular changes during malignant progression, we selected an index patient to obtain parallel tissue samples from the primary and metastatic tumors. In the metastatic tissue, 10 unique molecular alterations, including those in *TP53* and *APAF1*, were detected. In vitro functional experiments showed that *APAF1* depletion increased the tumor potency of cells expressing NAB2-STAT6 fusion protein under treatment with staurosporine. We found that TP53 immunopositivity (*P* = 0.006) and loss of APAF1 immunoreactivity (*P* < 0.001) were significantly associated with malignant SFTs. Our study suggests that dysfunction of *TP53* and *APAF1* leads to impaired apoptotic function, and eventually contributes toward malignant SFT transformation.

**Key messages:**

- We firstly found that the *TERT* promoter mutation was strongly associated with malignant SFTs (*P* = 0.003) and the representative 1b (*NAB2*ex4-*STAT6*ex2) or 2a (*NAB2*ex6-*STAT6*ex16) fusion variants similarly contribute to tumorigenicity.
- We also found that TP53 immunopositivity (*P* = 0.006) and loss of APAF1 immunoreactivity (*P* < 0.001) were significantly associated with malignant SFTs.
- Our study suggests that dysfunction of *TP53* and *APAF1* leads to impaired apoptotic function, and eventually contributes toward malignant SFT transformation.

**Electronic supplementary material:**

The online version of this article (10.1007/s00109-019-01815-8) contains supplementary material, which is available to authorized users.

## Introduction

Solitary fibrous tumors (SFTs) are uncommon mesenchymal tumors belonging to a group of fibroblastic/myofibroblastic tumors [1, 2]. In the past, they were considered as two distinct entities, namely SFT and hemangiopericytoma (HPC). However, because of the histological similarity and detection of the same *NAB2-STAT6* fusions, they are now considered as the same entity as per the current WHO classifications [3–5]. According to the current WHO classifications, SFTs are classified into two types, borderline and malignant, except for the meningeal tumors, which are classified as benign, borderline, and malignant [3–5]. However, histological parameters for classification, such as mitotic counts, pleomorphism, tumor necrosis, and cellularity, are incomplete for precisely determining the malignant potential [6, 7]. Most SFTs are considered non-malignant neoplasms and are usually treated by surgical resection [8]. A total of 15–20% of SFTs are generally aggressive, showing metastasis or recurrence and are difficult to treat [9, 10]. Chemotherapy plus bevacizumab, pazopanib, sunitinib, figitumumab, or bevacizumab has recently emerged as a promising potential therapeutic strategy for the treatment of advanced SFT [11, 12]. However, the management of SFTs in patients who had developed locally recurrent or metastatic cancer has been challenging.

The *NAB2-STAT6* fusion was first identified in two SFT cohorts by Robinson et al. and Chmielecki et al. through whole-exome sequencing (WES) studies [13, 14]. Subsequently, many groups have detected the *NAB2-STAT6* fusion in SFT/HPC by WES, transcriptome sequencing, and reverse transcription polymerase chain reaction (RT-PCR). A recent meta-analysis found that more than 40 *NAB2-STAT6* fusion variant types were present in up to 83% (452/546) of SFTs/HPCs, with *NAB2*ex6*-STAT6*ex16/17/18 and *NAB2*ex4*-STAT6*ex2/3 being the most frequent variants [15]. Interestingly, Barthelmess et al. suggested that *NAB2*ex6*-STAT6*ex16/17 was significantly associated with malignancy in SFTs/HPCs [16]. However, Tai et al., Chuang et al., and Yuzawa et al. reported that no fusion variants were associated with malignancy [17–19]. Therefore, the association between the fusion variants and malignant potentials is still unclear. In addition, there are few studies evaluating whether malignant SFTs have additional alterations as compared to that of non-malignant SFTs. Notably, recent reports have suggested that *TERT* promoter hot spot mutations are strongly associated with shorter disease-free survival, event-free survival, and high-risk clinicopathological characteristics in SFTs [6, 20, 21].

In this study, we investigated the incidence of *NAB2-STAT6* fusion transcript variants and the associations between *NAB2-STAT6* fusion variants or *TERT* promoter mutations and clinicopathological parameters in an SFT cohort. We performed a parallel WES to identify additional molecular changes in an index SFT case, which included a primary buttock sample and matching metastatic brain sample. We also explored whether the additional oncogenic mutations affected the malignancy of SFTs using stable cell lines expressing two representative fusion variants of NAB2-STAT6 by in vitro functional validation.

## Methods

### Patients

A total of 117 patients who had undergone surgical resection at Samsung Medical Center and diagnosed as SFT or HPC between 1995 and 2014 were included in our SFT cohort. Two pathologists reviewed all retrieved slides and confirmed the diagnosis with the morphology and the results of STAT6 and CD34 immunohistochemical staining. The 26 cases, with no available formalin-fixed paraffin-embedded (FFPE) tissue blocks or no STAT6 immunoreactivity, were excluded (Supplementary Fig. 1A). Then, we selected one case as the index case for WES. As a result, this study included 91 patients (men 53, women 38; median age 56 years, range 29–81 years) and one index case patient. Next, all cases were classified as non-malignant (including benign and borderline SFTs) or malignant SFT according to their mitotic activity. The cases with mitoses > 4/10 high-power fields (HPFs) were classified as malignant SFTs. Although meningeal SFT have different WHO criteria of the mitotic activity (> 5/10 HPFs), none of meningeal cases showed mitotic activity between > 4 and > 5 mitoses per 10 HPFs [5]. We were not able to use a recently proposed risk assessment model because we included meningeal SFTs in this study and this model was only validated in non-meningeal SFTs [6]. A 74-year-old female patient was selected as the index case of our study. This patient was diagnosed as SFT with a focal malignant change on the left buttock in August 2014, and showed a metastatic lesion in the brain in December 2014. The index patient with parallel tissue samples obtained from primary FFPE tissue and matched metastatic frozen tissue. This study was approved by the Institutional Review Board of Samsung Medical Center in Seoul, Korea (IRB file No. 2015–06-114).

### Cell lines and reagents

NIH3T3 cells were obtained from the Korean Cell Line Bank. The cells were cultured in Dulbecco’s Modified Eagle Medium (DMEM) (Thermo Fisher Scientific, Waltham, MA, USA) containing 10% fetal bovine serum (FBS) (Gibco, Grand Island, NY, USA) and 1% antibiotic–antimycotic (Gibco) at 37 °C and in 5% CO_2_.

### DNA and RNA extraction and cDNA synthesis

Genomic DNA from FFPE tissue was extracted using the QIAamp DNA Mini Kit (Qiagen, Hilden, Germany) according to the manufacturer’s instructions. Total RNA from the FFPE tissues was isolated using the RNeasy FFPE Kit (Qiagen) according to the manufacturer’s instructions. cDNA from the total RNA was synthesized using the SuperScript III First-Strand Synthesis System for RT-PCR (Invitrogen, Carlsbad, CA, USA).

### RT-PCR and PCR

*NAB2-STAT6* fusion variant-specific RT-PCR was performed using primer sets obtained from Barthelmess et al. (Supplementary Table 1A) [16]. RT-PCR was conducted using the Maxime RT-PCR premix kit (iNtRON Biotechnology, Seoul, Korea) with a temperature condition consisting of a pre-denaturation step at 95 °C for 5 min, followed by 50 cycles at 95 °C for 30 s, 57 °C for 45 s, and 72 °C for 1 min, and final extension step at 72 °C for 5 min. Amplified PCR products were confirmed by 1.5% agarose gel electrophoresis and purified for Sanger sequencing. To validate the mutations found in the index-case from the WES data, the genomic DNA of primary and metastatic tumors was used for PCR. The primer sets were designed using Primer3 and Primer-Blast (NCBI) (Supplementary Table 1B). PCR was performed at 95 °C for 10 min, followed by 40 cycles at 95 °C for 30 s, 55 °C for 45 s, and 72 °C for 30 s, and final extension step at 72 °C for 5 min. All PCR products were confirmed by Sanger sequencing.

To confirm *TERT* promoter mutations, PCR was conducted using PCR conditions and a primer set targeting regions surrounding two common TERT promoter hotspot mutations, -124C > T and -146C > T, for genomic DNA of an SFT cohort as previously described (Supplementary Table 1C) [22].

### Bisulfite sequencing

Genomic DNA was isolated from FFPE blocks using the QIAamp DNA Mini Kit (Qiagen, Hilden, Germany). Bisulfite conversion of DNA was performed using EZ DNA Methylation Kit (ZYMO Research, Irvine, CA, USA). The converted DNA samples were subjected to PCR amplification using a primer pair in a Furukawa et al.’ report (Supplementary Table 1D) [23].

### RNA-Seq and WES

RNA was isolated from fresh-frozen metastatic brain tissue (malignant tumor tissue) and its CDNA libraries were synthesized using the Nextera XT DNA Sample Prep Kit (Illumina, San Diego, CA, USA) and sequenced on a HiSeq 2500 using the 100-bp paired-end mode of the TruSeq Rapid PE Cluster kit and TruSeq Rapid SBS kit. For chimeric splicing junction analysis, GSNAP was used to perform paired-end-mode mapping of the reads on the pair of gene sequences involved in the fusion, without allowing any mismatch, indel, or splicing. DNA was prepared from a matched primary buttock tumor FFPE sample and fresh-frozen metastatic brain tissue of the index case patient. WES was performed using the TruSeq Exome Enrichment Kit (Illumina) and SureSelect Human All Exon Kit (Agilent Technologies, Santa Clara, CA, USA). Paired-end libraries were sequenced on an Illumina HiSeq 2000. Raw reads in FASTQ format from WES were aligned to the reference genome hg19 using the Burrows-Wheeler Aligner and duplicates were removed with Picard. WES data were analyzed using two INDEL calling algorithms, (1) GATK and, (2) SnpEff, following the guidelines provided in the user manuals. INDELs were called with each algorithm and variants were annotated using the ANNOVAR program.

### Immunohistochemistry (IHC)

Four-micrometer-thick sections from FFPE tissue blocks were cut with a microtome and routinely deparaffinized. The sections were incubated with 0.3% hydrogen peroxide. The antigen retrieval procedure was performed in 0.01 M of citrate buffer (pH 6.0) or Tris-EDTA Buffer (10 mM Tris, 1 mM EDTA, 0.03% Tween 20 (pH 9.0)) at 95 °C, and counterstaining was conducted with hematoxylin. The STAT6 antibody (Santa Cruz Biotechnology, Dallas, TX, USA, sc-621, 1:400 dilution) was used for STAT6 immunohistochemical staining. The CD34 antibody (Thermo Fisher Scientific, Inc., MA1-22646, 1:100 dilution) and Ki-67 antibody (Novocastra, Buffalo Grove, IL, USA, NCL-Li-Ki-67-MM1, 1:50 dilution) were used. The immunohistochemistry (IHC) for APAF1 was performed using an anti-APAF1 antibody (Sigma, St. Louis, MO, USA, PRS2015, 1:400 dilution) and IHC for TP53 was performed using the TP53 antibody (Vector Laboratories, Burlingame, CA, USA, VP-P958, 1:50 dilution). Loss of immunohistochemical reactivity for APAF1 was defined as no (0) or weak (1+) staining intensity of the tumor cells. Positive immunohistochemical reactivity for TP53 was defined as near complete absence of the immunoreactivity or positive immunoreactivity in more than 50% of the tumor cells.

### Construction of expression plasmids of fusion variants of *NAB2–STAT6*

For the generation of *NAB2*ex4-*STAT6*ex2 and *NAB2*ex6–*STAT6*ex16, *NAB2* cDNA was amplified using the pCDH1-*NAB2* lentiviral vector provided by Monika C. Wolkers. *STAT6* cDNA was generated from pCMV-*STAT6*-IRES-Neo (Addgene, Cambridge, MA, USA, Plasmid #35482). The PCR products were cloned into the N-terminal p3XFLAG-CMV-10 vector (Sigma-Aldrich, E7658). We confirmed the full sequence of wild-type *NAB2* and wild-type *STAT6* by the Sanger sequencing method. First-strand PCR was performed using the p3XFLAG-CMV vector containing two different wild-type fragments. The *NAB2* exon 4 and *NAB2* exon 6 fragments were synthesized with the following primer sets: forward, 3xFlag universal-F (ATGGACTACAAAGACCATGA) and reverse, *NAB2* e4_bpR (GGACTTGGAGGTTGCCTCTTGTTTCAGCTTCTTCA) and *NAB2* e6_bpR (CTATCTGTGGAGAGCCTGCGAGA GGTGGCTTCG). The *STAT6* exon 2 and *STAT6* exon 16 fragments were amplified with the following primer sets: forward, STAT6 e2-bpF (AAGCTGAAACAAGAGGCAACCTCCAAGTCCCAGAT) and STAT6 e16-bpF (AGCCACCTCTCGCAGGCTCTCCACAGATAGAGAACA), reverse, STAT6-R (TCACCAACTGGGGTTGGC). The second overlapping PCR was conducted using a mixture of two products as a template with the 3xFlag universal-F and STAT6-R primers. Full-length *NAB2*ex4–*STAT6*ex2 and *NAB2*ex6–*STAT6* ex16 variants were cloned into a gateway entry vector pCR8/GW/Topo (Invitrogen, K250020) and then subcloned into pLenti6.3/V5-DEST (Invitrogen, V53306). Full-length sequences of *NAB2-STAT6* fusion variants were validated by Sanger sequencing.

### Transient transfection and generation of stable cell lines expressing NAB2-STAT6 fusion variants

NIH3T3 cells were plated in a 60-mm dish (5 × 10^5^ cells) and then incubated in DMEM growth medium at 37 °C in 5% CO_2_. After 24 h, NIH3T3 cells were transfected with plasmids (10 μg) encoding the *NAB2-STAT6* fusion and pLenti empty vector using Lipofectamine LTX (Invitrogen) according to the manufacturer’s instructions. The pLenti6.3/NAB2-STAT6 expression vector was transfected into 293FT cells using the ViraPower Packaging Mix (Invitrogen, K497500) to produce lentivirus. After 48 h, lentivirus was harvested and transduced into NIH3T3 cells in the presence of 8 μg/mL of polybrene. DMEM complete medium was transferred after 48 h, and medium containing blasticidin (5 μg/mL) was replaced after 24 h. Cells were selected for 2 weeks using selective medium. Stable expression of NAB2-STAT6 was confirmed by qRT-PCR and Western blotting. Total RNA from the cells expressing *NAB2-STAT6* was isolated for qRT-PCR. qRT-PCR was conducted with SYBR Green PCR Master Mix (Applied Biosystems, Foster City, CA, USA, 4367659) using *NAB2-STAT6*-specific primer sets. qRT-PCR data was normalized to mouse GAPDH or mouse HPRT as reference genes using the ΔCt method. For Western blotting, protein lysates from cells expressing NAB2-STAT6 were separated by 10% SDS-PAGE and transferred to polyvinylidene fluoride membranes (Bio-Rad, Hercules, CA, USA). Western blotting was performed with the following antibodies: monoclonal anti-FLAG M2 antibody (Sigma-Aldrich, F3165, 1:2000) and GAPDH antibody (Santa Cruz, sc-25778, 1:2000).

### Apoptosis assay

Cell apoptosis was determined using the FITC Annexin V Apoptosis Detection Kit I (BD Biosciences, San Jose, CA, USA, 556547) following treatment with 1 μM of staurosporine (STS) (Sigma-Aldrich, S6942). Cell apoptosis was analyzed by an FACS Aria (BD Biosciences).

### Cell proliferation and migration assays

The cell proliferation assay was performed with the EZ-CYTOX cell proliferation kit (Daeil Lab Service, Seoul, Korea, EZ-1000) according to the manufacturer instructions. NIH3T3 cells expressing an empty vector or NAB2ex4–STAT6ex2 or NAB2ex6–STAT6ex16 were plated in 96-well plates (5 × 10^2^ cells/well). The 96-well plates were incubated with EZ-CYTOX reagent for 3 h at 37 °C after 1, 2, 3, 4, and 5 days. Absorbance was measured at 450 nm using a spectrophotometer. Twenty-four-well Transwell chambers (Corning Costar, Corning, NY, USA, #3422) with 8-μm polycarbonate membrane filters were used to determine cell migration ability. Next, 5 × 10^4^ cells of NIH3T3-empty and NIH3T3-encoding NAB2-STAT6 fusion variants were seeded into the upper chamber in DMEM without FBS. The lower chamber contained 700 μL of DMEM containing 10% FBS. The Transwell chamber was incubated at 37 °C in 5% CO_2_. After 24 h of incubation, non-migrating cells on the upper filter surface were removed with a cotton swab and migrated cells were stained with 0.5% crystal violet.

### Statistical analysis

Categorical variables were analyzed using the chi-square test or Fisher’s exact test. *P* values ≤ 0.05 (two-tailed) were used to establish statistical significance. All statistical analyses were conducted using SPSS version 21.0 (IBM SPSS, Armonk, NY, USA).

## Results

### Incidence of *NAB2-STAT6* fusion variants in SFT cohorts

A total of 117 cases were diagnosed as SFTs or HPC, and of them, 91 cases were evaluated (Supplementary Fig. 1A). We detected three variants of the *NAB2-STAT6* fusion transcript in 68 cases from the SFT cohort. The prevalence of the variant 1b (*NAB2*ex4-*STAT6*ex2) was 56% (*n* = 51), 2a (*NAB2*ex6-*STAT6*ex16) was 13% (*n* = 12), and 2b (*NAB2*ex6-*STAT6*ex17) was 6% (*n* = 5) (Supplementary Fig. 1A). The sequences in the other 23 cases could not be identified by RT-PCR using the five primer sets and were retained for the analysis of other *NAB2-STAT6* fusion variants. The three different fusion variants were reconfirmed in 68 cases (75%) by Sanger sequencing. The corresponding fusion junctions of the *NAB2-STAT6* variants are shown in Supplementary Fig. 1B.

### Associations of clinicopathological characteristics with *NAB2-STAT6* variants and *TERT* mutation status in SFTs

To investigate the associations between *NAB2-STAT6* variants and clinicopathological data, we analyzed the clinicopathological characteristics of 91 histologically confirmed SFT patients displaying different *NAB2-STAT6* variants (Table 1 and Supplementary Table 2). Variant 1b was significantly associated with pleural location (*P* < 0.001). Variants 2a/2b were significantly associated with meningeal location (*P* < 0.001). We found no significant association between any *NAB2-STAT6* variant and any other clinicopathological parameter.

**Table 1:** Associations of *NAB2–STAT6* variants with clinicopathological parameters in solitary fibrous tumors

	Case no.	*NAB2-STAT6*			*P* values
		1b	2a/2b	Not identified	
Gender	91				0.744
Male	53	28	11	14	
Female	38	23	6	9	
Age	91				0.119
< 56	52	33	10	9	
≥ 56	39	18	7	14	
Location	91				< 0.001
Meningeal	14	3	7	4	
Pleural	40	33	4	3	
Extrapleural	36	14	6	16	
Histologic subtypes	91				0.257
Non-malignant	72	39	14	19	
Malignant	19	12	3	4	
History of recurrence	91				0.921
Yes	10	6	2	2	
No	81	45	15	21	
History of metastasis	91				0.697
Yes	3	2	0	1	
No	88	49	17	22	
Survival	91				0.895
Yes	85	48	16	21	
No	6	3	1	2	

We wanted to evaluate whether SFTs progress to malignant transformation under *TERT* promoter mutations. The results were not available in 18 cases. In the remaining 73 SFT cases, only the -124C > T *TERT* promoter mutation was detected in seven cases (10%) and was strongly associated with malignant SFTs (*P* = 0.003) and the presence of necrosis (*P* = 0.036) (Supplementary Table 3, and Supplementary Fig. 2). However, the association between *TERT* promoter mutation and clinically aggressive behavior, which includes recurrence and/or metastasis, was not strong enough to show statistical significance (*P* = 0.205). All seven instances of *TERT* promoter mutations were discovered in older patients (≥ 56 years old) (Supplementary Table 3).

### Characterization of genomic alterations between non-malignant and malignant SFTs

**Fig. 1:**
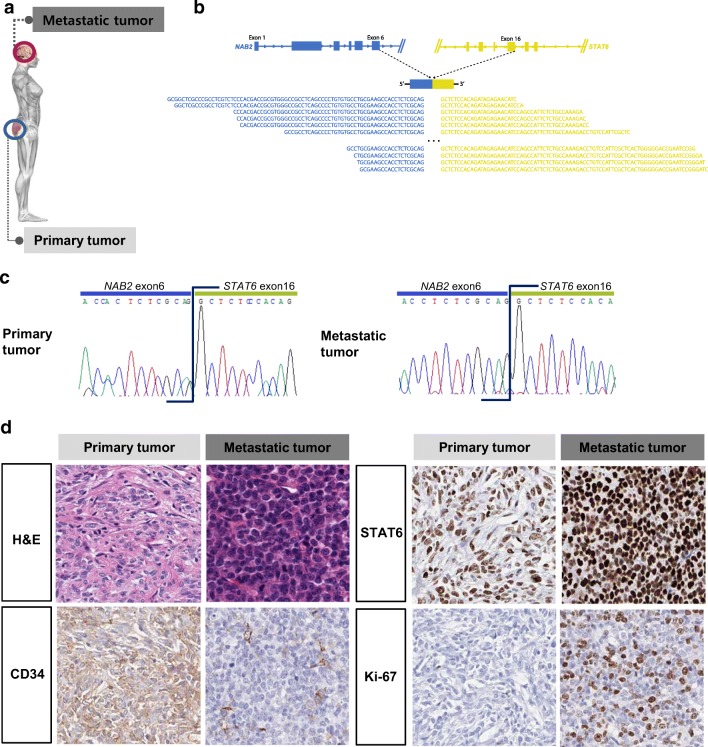
Characterization of the molecular and histopathological features of the index SFT case. **a** Diagram of the primary and metastatic sites of the index case. **b** A schematic pre-mRNAs of the fusion variant 2a from the RNA-sequencing experiment. Bottom sequences in black are the reads that map onto the chimeric exon-exon splicing junction. **c** The fusion variant 2a was confirmed by RT-PCR and Sanger sequencing. **d** Comparison of CD34, Ki-67, and STAT6 immunohistochemical staining and counterpart H&E staining in the primary and metastatic tissues

To identify additional oncogenic mutations in the malignant SFT, we selected an index patient to obtain parallel tissue samples from the primary and metastatic tumors. The index patient had a primary SFT in the buttock, and the primary tumor metastasized to the brain after 3 months (Fig. 1a). The primary tumor showed typical non-malignant SFT features in most areas, with a very focal area showing increased cellularity and mitotic activity. We selected a typical non-malignant SFT area from the primary case for molecular evaluation (Fig. 1d). The metastatic tumor presented as a totally histologic malignant SFT (Fig. 1d). We identified the same fusion variant type 2a in both the primary and metastatic tumors by RNA-sequencing, and RT-PCR, and Sanger sequencing (Fig. 1b, c). We evaluated the immunohistochemical expression of CD34 as a mesenchymal cell marker, STAT6 as an SFT diagnostic marker, and Ki-67 as a proliferation marker in the index case (Fig. 1d). The metastatic tumor cells expressed elevated levels of Ki-67, and STAT6, but decreased levels of CD34 relative to those in primary tumor cells (Fig. 1d). The *TERT* promoter hot spot mutations (-124C > T and -146C > T) were not detected in the index case (data not shown).

**Fig. 2:**
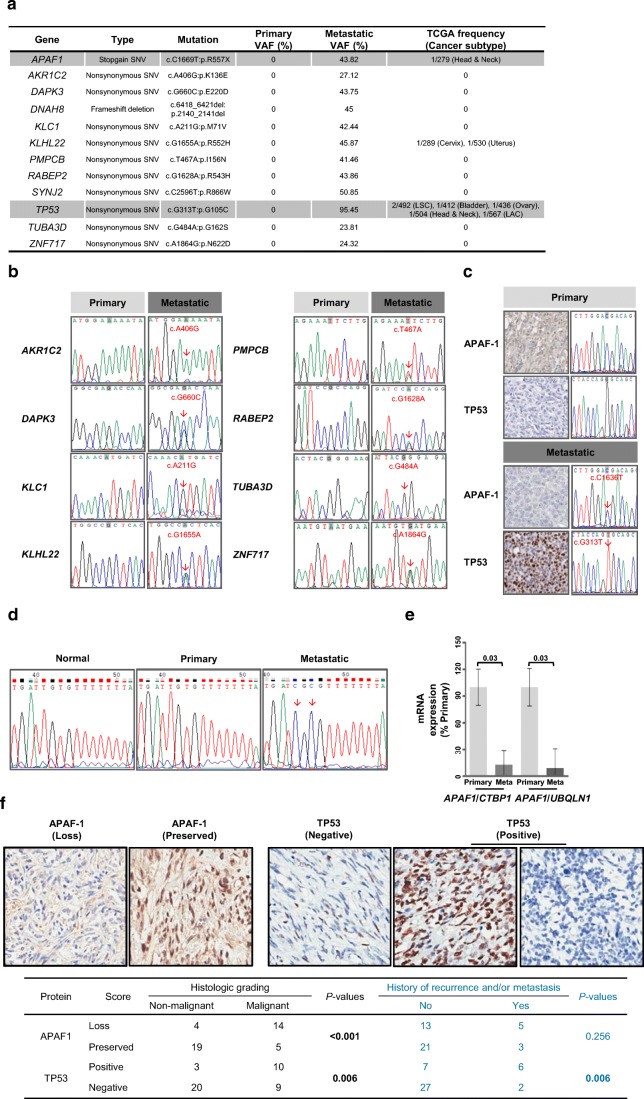
Characterization of genomic alterations between primary and metastatic tissues in the index SFT case. **a** Genes mutated only in the metastatic tissue and incidences of each mutation from TCGA cancer subtype. **b** The mutated genes were confirmed by Sanger sequencing in the primary and metastatic tissues of the index case. **c** Molecular changes and protein expression of *APAF1* and *TP53* were validated by Sanger sequencing and immunohistochemical assay, respectively. **d***APAF1* promoter DNA methylation on the SP1 binding motif was detected only in the metastatic tissues by bisulfite sequencing. **e** Reduction in *APAF1* mRNA was quantified in primary and metastatic tissues of the index case using qRT-PCR (*n* = 3; bar represents the SE, Student’s *t* test. **f** APAF1 and TP53 protein expression were monitored through IHC of tissues obtained from patients with SFTs. Comparisons of TP53 immunopositivity and loss of APAF1 immunoreactivity in non-malignant and malignant solitary fibrous tumors are shown

Next, we performed parallel WES on the primary tissue and matched metastatic tissue from this index case patient. These samples were sequenced to meet a minimum coverage of all targeted regions, with a minimum of > 81.1% exhibiting > 20× coverage. The primary samples contained 21,727 single-nucleotide variations and 14,453 small insertions and deletions (INDELs), while the metastatic samples contained 21,088 single-nucleotide variations and 14,375 INDELs (data not shown). Among all somatic variations, mutations were found only in the primary tumor. The synonymous mutations and unknown mutations with variant allele fractions < 20% were excluded from further analysis. Following this filtering, 12 genes were selected for re-sequencing (Sanger sequencing, primers shown in Supplementary Table 1B) (Fig. 2a–c). The corresponding mutations were confirmed in 10 genes, excluding *DNAH8* and *SYNJ2* (Supplementary Fig. 3). We then evaluated whether these 10 mutations had previously been reported in The Cancer Genome Atlas (TCGA) database. Mutations in *APAF1* (c. 1669C > T), *KLHL22* (c.1655G > A), and *TP53* (c.313G > T) were found in several cancer subtypes, and the *TP53* mutation was predicted to be pathogenic based on its FATHMM algorithm score (0.99) (Fig. 2a). We then focused on *APAF1*, which had a variant allele fraction (VAF) of 43.82% (expected for a heterozygous gain of a stop codon mutation), and *TP53*, which had a VAF of 95.45% (expected for a homozygous mutation) (Fig. 2a). The molecular changes and corresponding immunohistochemical expression for APAF1 and TP53 were evaluated using archived formalin-fixed paraffin-embedded (FFPE) samples. We confirmed the presence of the *APAF1* (c.1669C > T) and *TP53* (c.313G > T) mutations in FFPE samples of metastatic tumors by Sanger sequencing (Fig. 2c, right panel). Interestingly, APAF1 immunoreactivity was detected in the primary tumor but was reduced in the metastatic tumor. Conversely, TP53 protein expression was faintly detected in the sole focal areas of the primary tumors, while the metastatic tumors showed strong and diffuse immunoreactivity of TP53 (Fig. 2c, left panel).

We further screened for *APAF1* (c. 1669C > T in exon 12) and *TP53* (c.313G > T in exon 4) mutations in the 19 malignant SFT tissues. In addition, we tested for the previously reported mutations in *APAF1*, particularly focusing on exons 9–14 corresponding to the hinge region including exon 12, and in *TP53*, focusing on exons 5–8 corresponding to the reported hot spot areas responsible for the DNA-binding domain. *TP53* mutation analysis failed in 3 cases, and revealed *TP53* mutation in 6 (6/16, 37.5%) cases (including one c.313G > T, six c.742C > T, one c.818G > A, and one c.832C > T mutation) (Supplementary Tables 4 and 5).

*APAF1* is inactivated by DNA methylation in several cancers and leukemia [24–26]. The putative binding sites of known transcription factors, *TP53*, *SP1*, and *E2F*, have been identified in the *APAF1* promoter region [27, 28]. As *APAF1* was altered by a heterozygous mutation resulting in the gain of a stop codon in the index case, we assessed whether these DNA-binding motif sequences were methylated in the remaining allele. DNA methylation at the SP1 binding motif of the *APAF1* promoter was detected only in the metastatic tissues using bisulfite sequencing (Fig. 2d and Supplementary Table 1D). *APAF1* mRNA expression was also decreased in the metastatic tissues as compared to that in the primary tissue (Fig. 2e).

Next, we evaluated TP53 and APAF1 protein expression status in our cohort (19 malignant SFTs and 23 non-malignant SFTs). TP53 immunopositivity (*P* = 0.006) and loss of APAF1 immunoreactivity (*P* < 0.001) were significantly associated with the malignant SFTs (Fig. 2f). Clinically aggressive behavior, which includes known history of recurrence and/or metastasis, showed statistically significant association with TP53 immunopositivity (*P* = 0.006), but not with the loss of APAF1 immunoreactivity (*P* = 0.256) (Fig. 2f). However, the cases with loss of APAF1 expression showed a higher rate of recurrence and/or metastasis (27.8%, 5/18) as compared to those with intact APAF1 expression (12.5%, 3/24).

### In vitro functional validation of APAF1 as a driver of malignant transformation in SFT

**Fig. 3:**
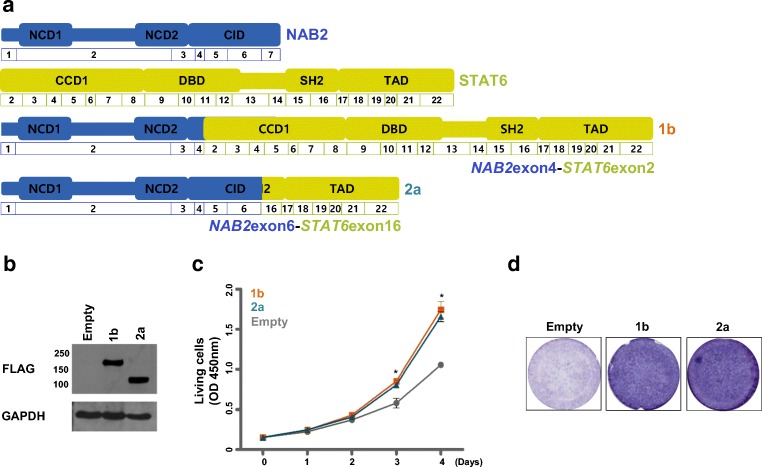
Comparison of representative 1b and 2a NAB2-STAT6 fusion variants’ roles on tumor progression. **a** Diagram of representative 1b or 2a *NAB2-STAT6* fusion variants. **b** The ectopic-expression of representative variant 1b or 2a fusion proteins was validated by immunoblotting. **c** The cell proliferation was evaluated using WST-1 assay in stable NIH-3T3 cell lines expressing representative variant 1b or 2a forms (gray; empty, orange; long, greenish blue; short) (*n* = 3; bar represents the SE, Student’s *t* test was performed, *P* values are presented in comparison with empty and variant 1b or 2a forms). **d** Migration assay was performed in the Transwell chamber. The images of crystal violet-stained cells expressing the indicated protein on the membranes are depicted

Based on the inactivation of APAF1 in the metastatic tissue of the index SFT case, we used in vitro functional studies to evaluate whether this inactivation acts as an additional oncogenic hit inducing malignant SFT. First, we generated stable NIH-3T3 cell lines expressing representative variant 1b or 2a NAB2-STAT6 fusions and empty control cells (Fig. 3a and b).

Next, we compared the tumorigenic functional effects of the 1b or 2a fusion variants. We conducted proliferation and migration assays using these stable NIH-3T3 cells. Expression of either variant 1b or 2a fusion protein increased cell proliferation (*P* < 0.001) and migration as compared to that in the control cells; however, there was no significant difference in tumorigenesis between cells expressing variant 1b and those expressing 2a (Fig. 3c and d).

**Fig. 4:**
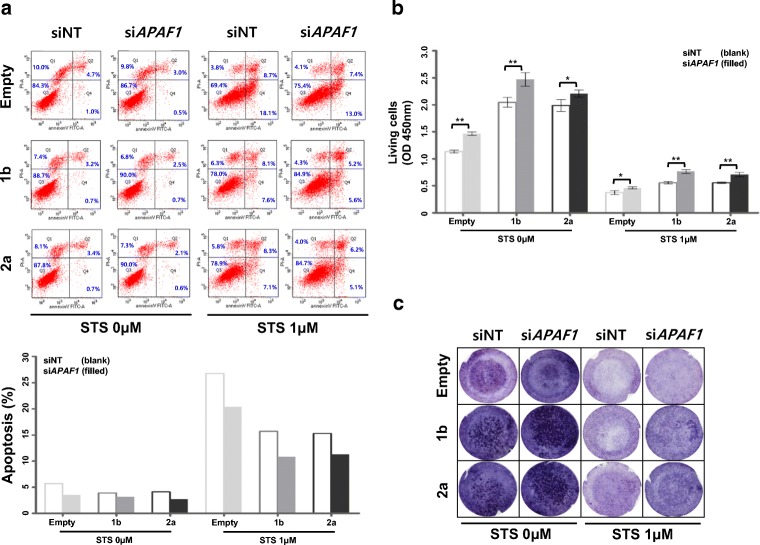
Evaluation of the potential role of *APAF1* depletion on malignant progression. **a** Upper panel: Effects of *APAF1* knockdown on apoptotic signaling was monitored via Annexin V assay using FACS, after treatment with 1 μM STS for 24 h. The numbers indicate the percentage of apoptotic cells in each quadrant. Lower panel: The values indicate the levels of apoptotic cells from the upper panel. **b** Increased cell viability of the indicated cells after STS treatment was evaluated by WST-1 assay (*n* = 3; bar represents the SE; *P < 0.05, **P < 0.05, Student’s *t* test. *P* values represent the indicated comparisons). **c** Migration assay was performed using Transwell chamber

APAF1 forms one of the central apoptosomes in the apoptotic regulatory pathway [29]. To investigate the role of APAF1 inactivation in malignant SFT progression, we evaluated whether *APAF1* depletion affects the viability, growth, and motility of stable cells expressing the 1b or 2a fusion variants under apoptotic conditions induced by treatment with staurosporine. *APAF1* depletion accelerated the inhibition of apoptosis in cells expressing variant 1b or 2a and in empty control cells as compared to that in the non-depleted cells (Fig. 4a). Moreover, as compared to the empty cells, the cells expressing the variant 1b or 2a fusion protein similarly exhibited reduced apoptosis (Fig. 4a). Notably, *APAF1* depletion increased the growth and motility of cells expressing variant 1b or 2a fusion protein relative to that in empty cells (Fig. 4b–c). Our findings suggest that APAF1 inactivation accelerates cell survival by inhibiting apoptosis and enhancing cell proliferation and migration. APAF1 inactivation may trigger an increase in SFT malignancy with similar potency in the common *NAB2-STAT6* fusion variant subtypes.

## Discussion

The goals of our study were to determine the incidence and clinical significance of *NAB2-STAT6* fusion transcript variants and *TERT* promoter mutations in the SFT cohort and explore the possibility of additional molecular alterations associated with malignant SFTs using an index patient.

We identified three variants of the *NAB2-STAT6* fusion transcript in 68 cases among 91 samples using RT-PCR and Sanger sequencing. The common fusion variants were variants 1b (*NAB2*ex4-*STAT6*ex2) in 51 (56%) SFTs, 2a (*NAB2*ex6-*STAT6*ex16) in 12 (13%) SFTs, and 2b (*NAB2*ex6-*STAT6*ex17) in 5 (6%) SFTs. In a review of the previous reports, our incidence rates were similar to those reported by Barthelmess et al. and Akaike et al. [16, 20]. In our cohort, variants 1b and 2a/2b were significantly associated with primary site tumors, which was also consistent with the previous studies (Supplementary Table 6) [13, 17, 20, 30, 31].

Most of the previous studies have reported that *NAB2-STAT6* fusion variants were not associated with malignant potentials [13, 17–20, 30, 32–37] (Supplementary Table 7). We also found no significant association between variants 1b or 2a/2b and malignant potentials. By performing in vitro functional validation, we first demonstrated that representative variant 1b or 2a fusion proteins demonstrated similar tumorigenicities. These results indicate that the representative 1b or 2a fusion variants contributed similarly to tumor cell proliferation and migration. Taken together, these results strongly suggest that the type of *NAB2-STAT6* fusion variant is not associated with the malignant behavior of SFTs; rather, malignant SFT may have additional oncogenic alterations in comparison with non-malignant SFT.

Recent reports have suggested that *TERT* promoter mutations are associated with old age, large tumor size, high-risk classifications, and short event-free survival [20, 21]. According to previous reports, the frequencies of *TERT* promoter mutations in SFT patients were 13% (5/40), 28% (26/94), 13% (4/31), and 20% (2/10) [21, 22, 38, 39]. Although we failed to find a significant association between *TERT* promoter mutation and recurrence, we found that the *TERT* promoter mutation (-124C > T) was strongly associated with malignant SFTs (*P* = 0.003) and the presence of necrosis (*P* = 0.036). These findings suggest that *TERT* promoter mutations promote aggressive tumor progression in SFTs. In addition, we found *TERT* promoter mutations only in elderly patients (≥ 56 years), which was also consistent with the findings of previous studies.

While *TERT* promoter mutations might correlate with the aggressiveness of SFT, a *TERT* promoter mutation was only found in a part of the malignant SFTs. To identify unspecified additional molecular changes in the malignant SFTs, we selected an index patient with a typical primary SFT in the buttock and metastatic brain tumor with an apparent malignant histological appearance. Through WES analysis followed by validation using Sanger sequencing, we identified 10 mutations in *APAF1*, *AKR1C2*, *DAPK3*, *KLC1*, *KLHL22*, *PMPCB*, *RABEP2*, *TP53*, *TUBA3D*, and *ZNF717* as unique molecular alterations in the metastatic SFT brain tissue. Among these 10 mutations, we especially focused on *APAF1* and *TP53* mutation.

*TP53* mutation has been already suggested to be associated with the malignant transformation of the SFTs in several previous reports [32, 40]. We also found statistically significant association between TP53 immunohistochemical positivity and malignant SFTs (*P* = 0.006) and clinical history of recurrence and/or metastasis (*P* = 0.006). In addition, we found *TP53* mutations in 41% (7/17) of the malignant SFTs in our cohort by molecular analysis. Interestingly, all found mutations (six c.742C > T, c.818G > A and c.832C > T of *TP53*) resulted in increased migration and proliferation [41]. Our findings suggest that *TP53* mutations are associated with malignant SFTs.

*TP53* is one of the most intensively investigated tumor suppressor genes, with hotspot mutations leading to loss of function. However, since the first report that the transduction of mutant TP53 protein into TP53-deficient cells enhanced tumorigenicity, numerous studies have demonstrated that a variety of *TP53* mutations can cause a neomorphic gain of function [41, 42]. Oncogenic *TP53* mutations are associated with inhibition of apoptosis by upregulating the transcription of various genes such as early growth response 1 (EGR1), a well-known upstream regulator of NAB2, and BCL2-associated athanogene (BAG1), a well-established blocker of apoptosis [43, 44]. It has been well established that wild-type TP53 protein is maintained at very low levels in most of the normal or cancer cells through strict regulation by MDM2, an E3 ubiquitin ligase targeting TP53, which forms a negative feedback loop [45, 46]. However, mutant TP53 proteins frequently accumulate at high levels in the tumor cells, and many reports have suggested that multiple events inhibit the degradation of mutant TP53 proteins during tumorigenesis, although mutant TP53 is mainly regulated by mechanisms consistent with those of the wild-type TP53 protein [47–49]. Interestingly, in the index case, the primary non-malignant tissue expressed minimal wild-type TP53 protein, while the metastatic brain tissue showed higher expression of mutant TP53 protein. Taken together, our data suggest that the mutant TP53 protein has an oncogenic function that may contribute to malignant progression.

In the metastatic brain SFT tissue of our index case, we detected *APAF1* (c.1669C > T) with a heterozygous mutation resulting in the gain of a stop codon. We found that APAF1 protein was expressed in the primary tumor, but was only minimally expressed in the mutant metastatic tumor. We also discovered that promoter DNA methylation inhibited *APAF1* mRNA expression, which might have resulted in the low expression of APAF1 protein observed in the metastatic brain tissue. Interestingly, we confirmed that the low expression of APAF1 protein is not a unique phenomenon in our index case by immunohistochemical staining of malignant SFTs in our cohort (14/21, 66.7%). We also revealed the statistically significant association between the low expression of APAF1 protein and the malignant SFTs (*P* = 0.002). The cases with low APAF1 expression also showed a higher rate of recurrence and/or metastasis (27.8%, 5/18) as compared to those with intact APAF1 expression (12.5%, 3/24). Unfortunately, the association was not strong enough to show statistical significance (*P* = 0.256) in this study. In addition, through functional validation using stable cells expressing variant 1b or 2a fusion forms, we demonstrated that APAF1 inactivation promotes cell survival through inhibition of apoptosis signaling and enhances tumorigenesis, which may trigger an increase in the tumor malignancy of SFT.

Our study has several limitations in finding a general mechanism of how SFTs turn malignant. Only one index case was included to identify additional molecular alterations in malignant SFTs and epigenetic changes in the whole genome were not determined. Each case may be unique in the process of evolving into malignant tumors. Our study confirmed events in one case, and although these results were only immunohistochemically elucidated in other cases, we think these findings will contribute to understanding the tumor evolution and malignant transformation of SFTs.

**Fig. 5:**
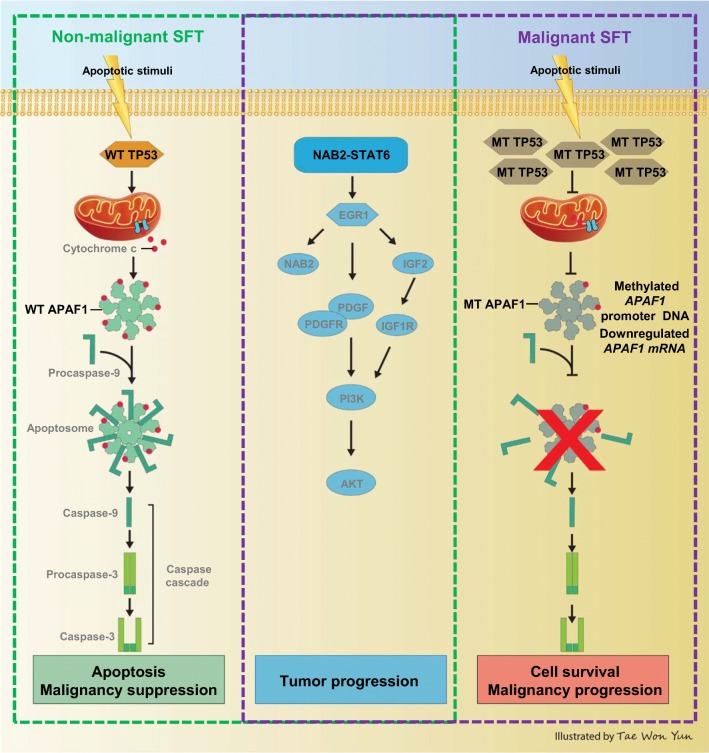
Model of TP53 and APAF1 roles in SFT with the NAB2-STAT6 fusion variant onto malignant transformation

Notwithstanding these limitations, based on our findings from the index case and those of Robinson et al., we suggest a model in which NAB2-STAT6 constitutively activates tumor proliferation and migration via EGR1 pathway under control of intact apoptosis signaling in non-malignant SFT. In contrast, the stabilized mutant TP53 and inactivated APAF1 with impaired apoptotic function trigger additional malignant features in malignant SFTs (Fig. 5) [13].

## Electronic supplementary material

Supplementary Figure 1 **(A)** Flow chart of the incidences of *NAB2-STAT6* fusion variants in our cohort. The variant types were confirmed by RT-PCR and Sanger sequencing. **(B)** The three representative *NAB2-STAT6* fusion variants were confirmed by RT-PCR and Sanger sequencing. (PPTX 71 kb)

Supplementary Figure 2 The *TERT* promoter mutation (-124C>T) was validated by Sanger sequencing in the malignant tissues. (PPTX 44 kb)

Supplementary Figure 3 The mutations of *DNAH8* and *SYNJ2* were not confirmed in the metastatic tissues. (PPTX 58 kb)

ESM 1 (XLSX 21 kb)

ESM 2 (DOCX 62 kb)

## Abbreviations

*SFT*: Solitary fibrous tumors
*HPC*: Hemangiopericytoma
*WES*: Whole-exome sequencing
*FFPE*: Formalin-fixed paraffin-embedded
*HPF*: High-power field
*IHC*: Immunohistochemistry
*INDEL*: Insertions and deletions
