# Supplementary material for: Molecular changes in solitary fibrous tumor progression
Source: J Mol Med (Berl). 2019 Jul 18;97(10):1413–25. doi: 10.1007/s00109-019-01815-8 (PMC6746689; doi:10.1007/s00109-019-01815-8)
Supplement: Supplementary file 1 — (A) Flow chart of the incidences of NAB2-STAT6 fusion variants in our cohort. The variant types were confirmed by RT-PCR and Sanger sequencing. (B) The three representative NAB2-STAT6 fusion variants were confirmed by RT-PCR and Sanger sequencing. (PPTX 71 kb) [file 109_2019_1815_MOESM1_ESM.pptx]

## Slide 1
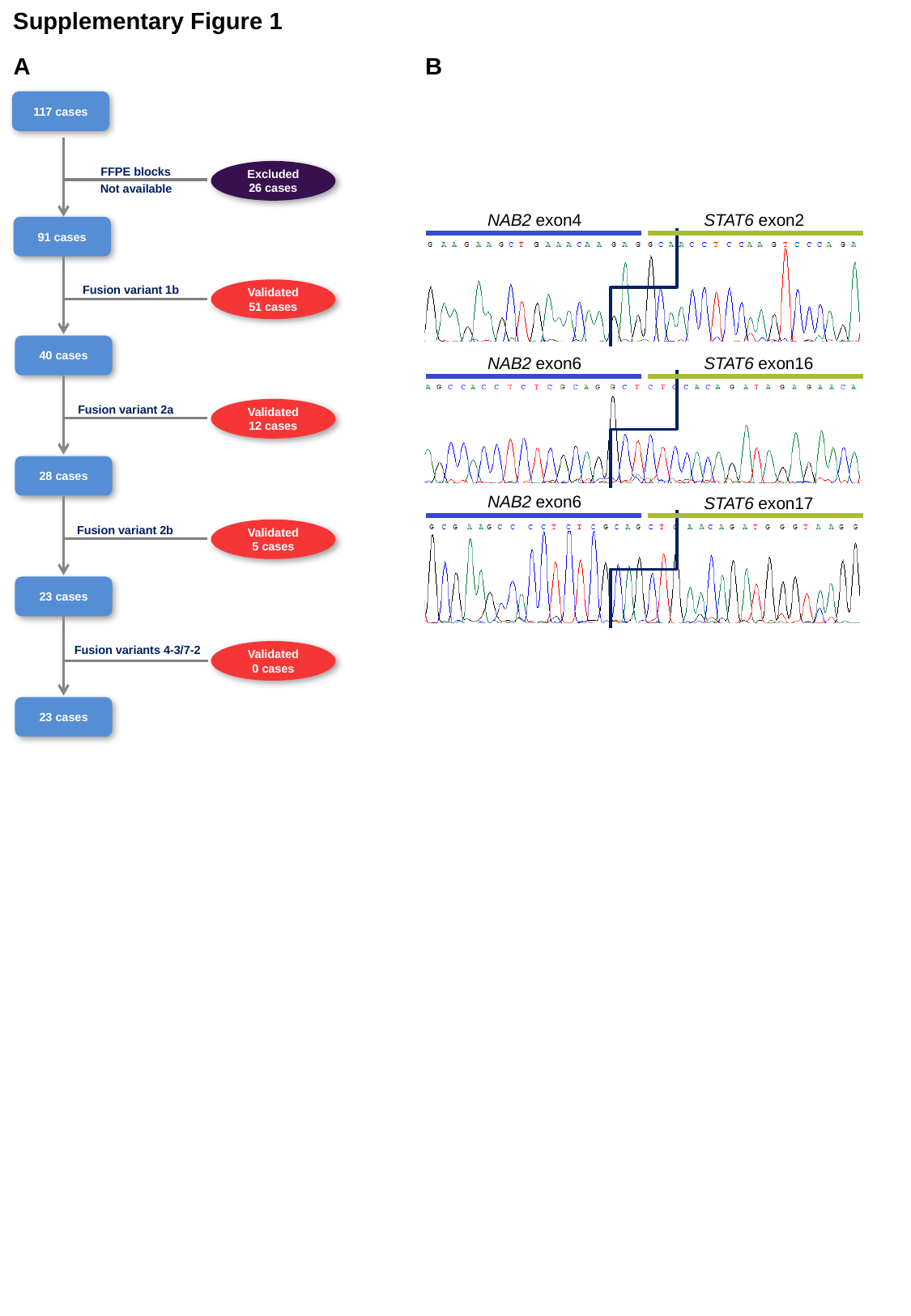

Supplementary Figure 1
B
A
117 cases
FFPE blocks
Excluded
26 cases
Not available
91 cases
Fusion variant 1b
Validated
51 cases
40 cases
Fusion variant 2a
Validated
12 cases
28 cases
Fusion variant 2b
Validated
5 cases
23 cases
Fusion variants 4-3/7-2
Validated
0 cases
23 cases
STAT6 exon2
NAB2 exon4
NAB2 exon6
STAT6 exon16
NAB2 exon6
STAT6 exon17
