# Supplementary figures and images for: Molecular changes in solitary fibrous tumor progression
Source: J Mol Med (Berl). 2019 Jul 18;97(10):1413–25. doi: 10.1007/s00109-019-01815-8 (PMC6746689; doi:10.1007/s00109-019-01815-8)

## Slide 1
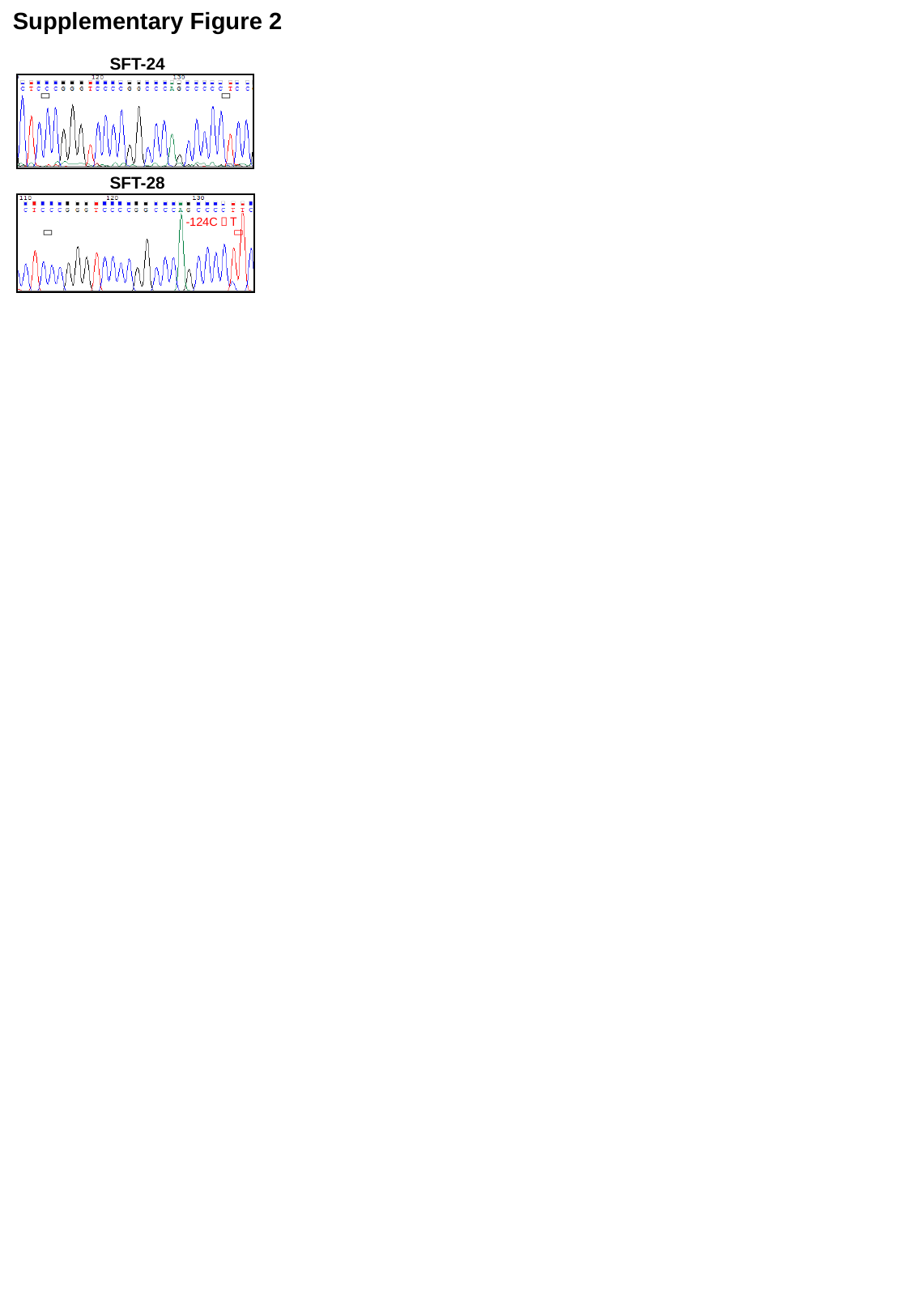

Supplementary Figure 2
SFT-24


SFT-28
-124C＞T



Supplement: Supplementary file 2 — The TERT promoter mutation (-124C>T) was validated by Sanger sequencing in the malignant tissues. (PPTX 44 kb) [file 109_2019_1815_MOESM2_ESM.pptx]

## Slide 1
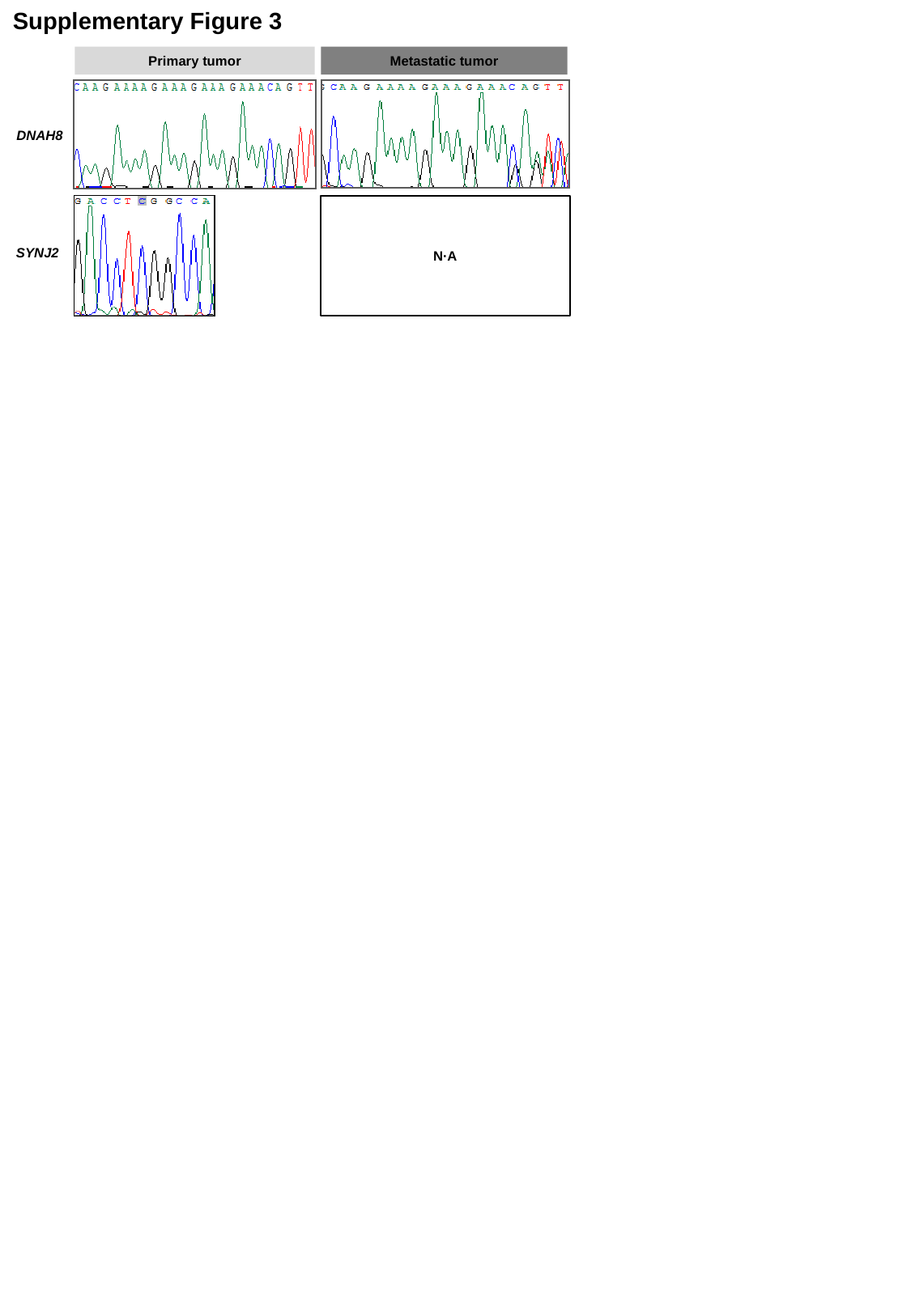

Supplementary Figure 3
Primary tumor
Metastatic tumor
DNAH8
N·A
SYNJ2

Supplement: Supplementary file 3 — The mutations of DNAH8 and SYNJ2 were not confirmed in the metastatic tissues. (PPTX 58 kb) [file 109_2019_1815_MOESM3_ESM.pptx]
