# Supplementary material for: Molecular changes in solitary fibrous tumor progression
Source: J Mol Med (Berl). 2019 Jul 18;97(10):1413–25. doi: 10.1007/s00109-019-01815-8 (PMC6746689; doi:10.1007/s00109-019-01815-8)
Supplement: Supplementary file 5 — (DOCX 62 kb) [file 109_2019_1815_MOESM5_ESM.docx]

**Supplementary Table 2~~5~~. Association of *NAB2-STAT6* variants with histological risk factors**

|  |  | ***NAB2-STAT6*** | | | | |  |
| --- | --- | --- | --- | --- | --- | --- | --- |
|  | **Case No.** | **1b** | **2a/2b** | | | **Not Identified** | ***P*-values** |
| **Cellularity** | 91 |  | |  |  | | 0.419 |
| Low | 11 | 5 | | 3 | 3 | |  |
| Moderate | 50 | 32 | | 6 | 12 | |  |
| Marked | 30 | 14 | | 8 | 8 | |  |
|  |  |  | |  |  | |  |
| **~~Mitotic activity~~** | ~~91~~ |  | |  |  | | ~~0.485~~ |
| ~~≤4~~ | ~~72~~ | ~~39~~ | | ~~14~~ | ~~19~~ | |  |
| ~~10 ≥ and 4<~~ | ~~11~~ | ~~6~~ | | ~~1~~ | ~~4~~ | |  |
| ~~>10~~ | ~~8~~ | ~~6~~ | | ~~2~~ | ~~0~~ | |  |
|  |  |  | |  |  | |  |
| **Pleomorphism** | 91 |  | |  |  | | 0.82 |
| Mild | 24 | 16 | | 1 | 7 | |  |
| Moderate | 48 | 28 | | 9 | 11 | |  |
| Marked | 19 | 7 | | 7 | 5 | |  |
|  |  |  | |  |  | |  |
| **Necrosis** | 91 |  | |  |  | | 0.386 |
| Absent | 81 | 45 | | 14 | 22 | |  |
| Present | 10 | 6 | | 3 | 1 | |  |

**Supplementary Table 3~~6~~. Associations of the *TERT* mutation with histopathological parameters and histological risk factors**

|  |  | ***TERT* mutation(-124 C**＞**T)** | | |  | |
| --- | --- | --- | --- | --- | --- | --- |
|  | **Case no.** | **Wild type** | **Mutant type** | **N.A** | | ***P*-values** |
| **Histologic subtypes** | 91 |  |  |  | **0.003~~2~~** | |
| Non-malignant | 72~~1~~ | 53~~2~~ | 2 | 17 |  | |
| Malignant | 19~~20~~ | 13~~4~~ | 5 | 1 |  | |
|  |  |  |  |  |  | |
| **Age** | 91 |  |  |  | **0.008** | |
| < 56 | 40 | 35 | 0 | 5 |  | |
| ≥ 56~~5<~~ | 51 | 31 | 7 | 13 |  | |
| ~~55≥~~ | ~~40~~ | ~~35~~ | ~~0~~ | ~~5~~ |  | |
|  |  |  |  |  |  | |
| **Cellularity** | 91 |  |  |  | 0.616 | |
| Low | 11 | 4 | 0 | 7 |  | |
| Moderate | 49 | 39 | 3 | 7 |  | |
| Marked | 31 | 23 | 4 | 4 |  | |
|  |  |  |  |  |  | |
| **~~Mitotic activity~~** | ~~91~~ |  |  |  | **~~0.024~~** | |
| ~~≤4~~ | ~~72~~ | ~~53~~ | ~~2~~ | ~~17~~ |  | |
| ~~10 ≥ and 4<~~ | ~~13~~ | ~~8~~ | ~~4~~ | ~~1~~ |  | |
| ~~>10~~ | ~~6~~ | ~~5~~ | ~~1~~ | ~~0~~ |  | |
|  |  |  |  |  |  | |
| **Pleomorphism** | 91 |  |  |  | 0.667 | |
| Mild | 24 | 18 | 1 | 5 |  | |
| Moderate | 48 | 32 | 5 | 11 |  | |
| Marked | 19 | 16 | 1 | 2 |  | |
|  |  |  |  |  |  | |
| **Necrosis** | 91 |  |  |  | **0.036** | |
| Absent | 81 | 60 | 4 | 17 |  | |
| Present | 10 | 6 | 3 | 1 |  | |
|  |  |  |  |  |  | |
| **Location** | 91 |  |  |  | 0.263 | |
| Meningeal | 15 | 13 | 1 | 1 |  | |
| Pleural | 40 | 25 | 5 | 10 |  | |
| Extrapleural | 36 | 28 | 1 | 7 |  | |
|  |  |  |  |  |  | |
| **Recurrence and/or Metastasis** | 91 |  |  |  | 0.205 | |
| No | 80 | 59 | 5 | 16 |  | |
| Yes | 11 | 7 | 2 | 2 |  | |

**Supplementary Table 4~~7~~. The data of *APAF1* mutations and protein expression in malignant patients**

**
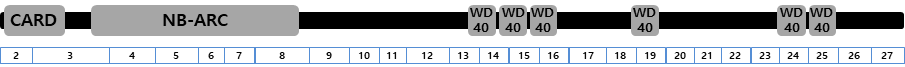
**

| **Malignant patient** | ***APAF1* exon12**  **(c. C1669T)** | ***APAF1* exon9** | ***APAF1* exon10** | ***APAF1* exon11** | ***APAF1* exon12** | ***APAF1* exon13** | ***APAF1* exon14** | **APAF1 expression** |
| --- | --- | --- | --- | --- | --- | --- | --- | --- |
| SFT2 | N/A | N/A | N/A | N/A | N/A | N/A | N/A | Preserved |
| SFT10 | WT | WT | WT | WT | WT | WT | WT | Loss |
| SFT13 | WT | WT | WT | WT | WT | WT | WT | Loss |
| SFT21 | N/A | WT | WT | WT | WT | WT | WT | Loss |
| SFT28 | WT | WT | WT | WT | WT | WT | WT | Loss |
| SFT29 | WT | WT | WT | WT | WT | N/A | WT | Preserved |
| SFT48 | WT | WT | WT | WT | WT | WT | WT | Loss |
| SFT50 | WT | WT | WT | WT | WT | WT | WT | Loss |
| SFT63 | WT | WT | N/A | N/A | WT | N/A | N/A | Preserved |
| SFT68 | WT | WT | WT | WT | WT | WT | WT | Loss |
| ~~SFT83~~ | ~~WT~~ | ~~WT~~ | ~~WT~~ | ~~WT~~ | ~~WT~~ | ~~WT~~ | ~~WT~~ | ~~Preserved~~ |
| SFT84 | WT | WT | WT | WT | WT | WT | WT | Loss |
| SFT85 | WT | WT | WT | WT | WT | WT | WT | Loss |
| SFT87 | N/A | WT | WT | WT | WT | N/A | WT | Loss |
| SFT95 | WT | WT | WT | WT | WT | WT | WT | Preserved |
| SFT99 | WT | WT | WT | WT | WT | WT | WT | Preserved |
| SFT107 | WT | WT | WT | WT | WT | WT | WT | Loss |
| SFT109 | WT | WT | WT | WT | WT | WT | WT | Loss |
| SFT114 | WT | WT | WT | WT | WT | WT | WT | Loss |
| SFT117 | WT | WT | WT | WT | WT | WT | WT | Loss |

**Supplementary Table 5~~8~~. The data of *TP53* mutations and protein expression in malignant patients**

**
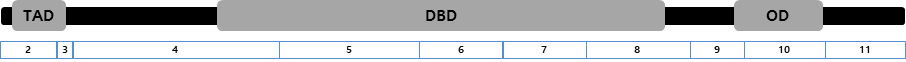
**

| **Malignant patient** | ***TP53***  **exon4**  **(c.G313T)** | ***TP53* exon5** | ***TP53* exon6** | ***TP53* exon7** | ***TP53* exon8** | **TP53 expression** |
| --- | --- | --- | --- | --- | --- | --- |
| SFT2 | N/A | N/A | N/A | N/A | N/A | Positive |
| SFT10 | WT | WT | N/A | WT | WT | Negative |
| SFT13 | WT | WT | WT | c.742C>T  (p.R248W) | c.832C>T (p.P278S) | Positive |
| SFT21 | N/A | N/A | N/A | N/A | N/A | Negative |
| SFT28 | WT | WT | WT | WT | WT | Negative |
| SFT29 | WT | N/A | N/A | N/A | N/A | Negative |
| SFT48 | WT | WT | WT | WT | WT | Negative |
| SFT50 | WT | WT | WT | WT | WT | Positive |
| SFT63 | c.313G>T  (p.G105C) | WT | WT | c.742C>T  (p.R248W) | N/A | Positive |
| SFT68 | WT | WT | WT | c.742C>T  (p.R248W) | WT | Positive |
| ~~SFT83~~ | ~~WT~~ | ~~WT~~ | ~~WT~~ | ~~c.742C>T~~  ~~(p.R248W)~~ | ~~WT~~ |  |
| SFT84 | WT | WT | WT | c.742C>T  (p.R248W) | WT | Positive |
| SFT85 | WT | WT | WT | WT | WT | Negative |
| SFT87 | N/A | WT | WT | WT | N/A | Positive |
| SFT95 | WT | WT | WT | c.742C>T  (p.R248W) | WT | Positive |
| SFT99 | WT | WT | WT | WT | WT | Negative |
| SFT107 | WT | WT | WT | WT | c.818G>A (p.R273H) | Positive |
| SFT109 | WT | WT | WT | WT | WT | Negative |
| SFT114 | WT | WT | WT | WT | WT | Positive |
| SFT117 | WT | WT | WT | WT | WT | Negative |

**Supplementary Table 6. Association of *NAB2-STAT6* variants with tumor locations obtained from previous reports**

| Reference | N | Meningeal | | |  | Pleural | | |  | Extrapleural | | |  | *P* |
| --- | --- | --- | --- | --- | --- | --- | --- | --- | --- | --- | --- | --- | --- | --- |
|  |  | 4-2/3/4 | 6-16/17/18 | Etc |  | 4-2/3/4 | 6-16/17/18 | Etc |  | 4-2/3/4 | 6-16/17/18 | Etc |  |  |
| This study | 91 | 3 | 7 |  |  | 33 | 4 |  |  | 14 | 6 |  |  | <0.001 |
| Dagrada *et al*, 2015 | 21 |  | 1 |  |  |  | 1 | 1 |  | 2 | 11 | 5 |  |  |
| Barthelmess *et al*, 2014 | 52 |  |  |  |  | 25 | 0 | 1 |  | 2 | 11 | 9 |  | <0.001 |
| Akaike *et al*, 2015 | 40 | 2 | 1 | 1 |  | 19 | 2 | 4 |  | 0 | 4 | 7 |  | <0.001 |
| Robinson *et al*, 2013 | 51 | 1 | 12 | 1 |  | 3 | 3 | 2 |  | 0 | 23 | 6 |  | 0.009 |
| Fritchie *et al*, 2016 | 30 | 7 | 13 | 10 |  |  |  |  |  |  |  |  |  |  |
| Vogel *et al*, 2014 | 26 |  |  |  |  | 9 | 0 | 0 |  | 2 | 4 | 11 |  | <0.001 |
| Mohajeri *et al*, 2013 | 37 | 1 | 1 | 0 |  | 8 | 0 | 0 |  | 10 | 8 | 9 |  | 0.11 |
| Chuang *et al*, 2016 | 37 |  |  |  |  |  |  |  |  | 8 | 13 | 9 |  |  |
| Huang *et al*, 2016 | 34 |  |  |  |  | 29 | 5 |  |  |  |  |  |  |  |
| Kao *et al*, 2016 | 19 |  |  |  |  |  |  |  |  | 5 | 8 | 6 |  |  |
| Tai *et al*, 2015 | 73 | 2 | 17 |  |  | 23 | 5 |  |  | 9 | 17 |  |  | <0.001 |
| Yuzawa *et al*, 2016 | 17 | 3 | 7 | 7 |  |  |  |  |  |  |  |  |  |  |
| Guseva *et al*, 2016 | 10 | 1 | 2 | 1 |  | 2 |  |  |  | 1 | 2 | 1 |  | 0.817 |

**Supplementary Table 7~~9~~. Association of *NAB2-STAT6* variants with histological subtypes obtained from previous reports**

|  | **Reference** | **N** | |  | | **Non-malignant** | | | | | | |  | **Malignant** | | | | | | |  | ***P*** |  |
| --- | --- | --- | --- | --- | --- | --- | --- | --- | --- | --- | --- | --- | --- | --- | --- | --- | --- | --- | --- | --- | --- | --- | --- |
|  |  |  |  |  |  | **4-1/2/3/4** | | | **6-16/17/18** | **Etc.** | | |  | **4-1/2/3/4** | | | **6-16/17/18** | **Etc.** | | |  |  |  |
| 1 | This study | | 91 | |  | | 38 | 14 | | | 19 |  | | | 13 | 3 | | | 4 |  | | 0.659 | |
| 2 | Dagrada *et al*, 2015 | 24 | |  | | 2 | | | 7 | 7 | | |  | 2 | | | 4 | 2 | | |  | 0.593 |  |
| 3 | Barthelmess  *et al*, 2014 | 52 | |  | | 23 | | | 4 | 14 | | |  | 4 | | | 7 | 0 | | |  | <0.001 |  |
| 4 | Akaike *et al*, 2015 | 40 | |  | | 15 | | | 3 | 4 | | |  | 6 | | | 4 | 8 | | |  | 0.083 |  |
| 5 | Robinson *et al*, 2013 | 51 | |  | | 2 | | | 22 | 5 | | |  | 1 | | | 16 | 5 | | |  | 0.850 |  |
| 6 | Fritchie *et al*, 2016 | 30 | |  | | 5 | | | 10 | 7 | | |  | 2 | | | 3 | 3 | | |  | 0.925 |  |
| 7 | Vogel *et al*, 2014 | 19 | |  | | 4 | | | 3 | 9 | | |  | 2 | | | 0 | 1 | | |  | 0.332 |  |
| 8 | Mohajeri *et al*, 2013 | 44 | |  | | 16 | | | 4 | 15 | | |  | 1 | | | 5 | 3 | | |  | 0.010 |  |
| 9 | Chuang *et al*, 2016 | 25 | |  | | 7 | | | 12 | | | |  | 1 | | | 5 | | | |  | 0.356 |  |
| 10 | Huang *et al*, 2016 | 34 | |  | | 24 | | | 3 | 0 | | |  | 5 | | | 2 | 0 | | |  | 0.245 |  |
| 11 | Kao *et al*, 2016 | 36 | |  | | 4 | | | 7 | 20 | | |  | 1 | | | 1 | 3 | | |  | 0.913 |  |
| 12 | Tai *et al*, 2015 | 73 | |  | | 30 | | | 32 | | | |  | 4 | | | 7 | | | |  | 0.461 |  |
| 13 | Yuzawa *et al*, 2016 | 15 | |  | | 3 | | | 6 | 4 | | |  | 0 | | | 0 | 2 | | |  | 0.117 |  |
| 14 | Guseva *et al*, 2016 | 8 | |  | | 2 | | | 3 | 1 | | |  | 0 | | | 1 | 1 | | |  | 0.513 |  |
